# Competence in *Streptococcus pneumoniae* Is a Response to an Increasing Mutational Burden

**DOI:** 10.1371/journal.pone.0072613

**Published:** 2013-08-13

**Authors:** Alyssa L. Gagne, Kathleen E. Stevens, Marco Cassone, Amit Pujari, Olufunke E. Abiola, Diana J. Chang, Michael E. Sebert

**Affiliations:** 1 Division of Infectious Diseases, Children’s Hospital of Philadelphia, Philadelphia, Pennsylvania, United States of America; 2 School of Engineering and Applied Science, University of Pennsylvania, Philadelphia, Pennsylvania, United States of America; 3 School of Arts and Sciences, University of Pennsylvania, Philadelphia, Pennsylvania, United States of America; 4 Department of Pediatrics, University of Pennsylvania Perelman School of Medicine, Philadelphia, Pennsylvania, United States of America; University of Kansas Medical Center, United States of America

## Abstract

Competence for genetic transformation in *Streptococcus pneumoniae* has previously been described as a quorum-sensing trait regulated by a secreted peptide pheromone. Recently we demonstrated that competence is also activated by reduction in the accuracy of protein biosynthesis. We have now investigated whether errors upstream of translation in the form of random genomic mutations can provide a similar stimulus. Here we show that generation of a mutator phenotype in *S. pneumoniae* through deletions of *mutX*, *hexA* or *hexB* enhanced the expression of competence. Similarly, chemical mutagenesis with the nucleotide analog dPTP promoted development of competence. To investigate the relationship between mutational load and the activation of competence, replicate lineages of the *mutX* strain were serially passaged under conditions of relaxed selection allowing random accumulation of secondary mutations. Competence increased with propagation in these lineages but not in control lineages having wild-type *mutX*. Resequencing of these derived strains revealed between 1 and 9 single nucleotide polymorphisms (SNPs) per lineage, which were broadly distributed across the genome and did not involve known regulators of competence. Notably, the frequency of competence development among the sequenced strains correlated significantly with the number of nonsynonymous mutations that had been acquired. Together, these observations provide support for the hypothesis that competence in *S. pneumoniae* is regulated in response to the accumulated burden of coding mutations in the bacterial genome. In contrast to previously described DNA damage response systems that are activated by physical lesions in the chromosome, this pneumococcal pathway may represent a unique stress response system that monitors the coding integrity of the genome.

## Introduction

Natural competence for genetic transformation is a widespread phenomenon found in diverse groups of bacteria, but the primary function of this trait has remained uncertain [1], [2]. Proposals regarding the role of competence have centered on the potential for newly acquired DNA to repair genetic lesions, the ability of such DNA to spread novel traits via horizontal gene transfer and the role of extracellular nucleic acids as a nutritional resource. The capacity of competence to improve the fitness of a bacterial population through recombination, however, has been questioned based on the concern that DNA available in the extracellular pool is likely to contain on average an excess of deleterious mutations reflecting an origin from the selective death of organisms that were less fit [3]. The observation that DNA damage does not induce competence in either *Haemophilus influenzae* or *Bacillus subtilis* has been seen as additional evidence against a primary repair function for competence in these organisms [4].

However, in other bacteria—including *Streptococcus pneumoniae* [5], *Helicobacter pylori* [6] and *Legionella pneumophila* [7]—competence has now been shown to be induced by the DNA crosslinking agent mitomycin C. Competence in the respiratory tract pathogen *S. pneumoniae* (aka the pneumococcus) is furthermore accompanied by a behavior described as fratricide wherein competent cells lyse other pneumococci [8]. This process of active bacterial predation has the potential to circumvent the problem of poor quality DNA in the extracellular pool and may thereby improve the ability of transformation to increase fitness.

Competence in *S. pneumoniae* is triggered by a secreted peptide pheromone known as the competence-stimulating peptide (CSP), which accumulates outside the cell and activates signaling by the ComDE two-component system [9]. This pathway has been considered an example of the peptide-mediated quorum sensing that is characteristic of gram-positive organisms, but subsequent findings have suggested that the system may not function specifically to monitor bacterial cell density [10]. In addition to induction by mitomycin C, the pneumococcal competence system is activated by certain antibiotics including the translation inhibitors streptomycin and kanamycin [5], generating a pattern of regulation that has been interpreted as a general stress response [10]. We recently demonstrated, however, that the induction of competence by streptomycin and kanamycin was specifically due to the increased frequency of decoding errors made by the ribosomes of cells treated with subinhibitory concentrations of these antibiotics [11]. Conversely, spontaneous competence was prevented by reducing the frequency of translational errors below the basal level [11]. These findings suggested that, rather than representing a general stress response, the pneumococcal competence pathway functions to monitor the accuracy of protein biosynthesis.

We were then interested in determining whether errors further upstream in the processes of replicating genetic information and accurately converting that information into finished proteins could have a similar effect. We therefore have examined the impact on competence of increasing the rates of errors during transcription and DNA replication. Although we did not envision that transformation would serve as an adaptive downstream response to the specific stress of transcriptional errors, induction of competence by this stimulus would support a broader model in which competence may be triggered by errors that reduce the accuracy of protein synthesis at different stages in cellular processing. We have proposed that this activation may result from the effects on competence regulators of proteases that also recognize and degrade unfolded proteins [11], [12]. This model is described further in the Discussion and has received preliminary support through our work characterizing the pneumococcal HtrA protease [12]. It also appeared possible that the broader pneumococcal competence response, which includes the induction of cellular proteases and chaperones in addition to genetic transformation [13], may serve an adaptive role in the setting of transcriptional errors that impair the ability of encoded proteins to fold properly.

Of particular interest was whether small increases in the genomic mutational load (i.e., degeneration of the information content of the genome rather than simply physical damage to the DNA) would affect the expression of competence. This question was addressed using several independent methods to introduce low levels of random mutations into the genome and then assaying the impact of these changes on competence. These methods included chemical mutagenesis with a nucleotide analog, utilization of mutator strains, and the development of a set of serial transfer lineages characterized by the random accumulation of mutations during laboratory passage. Importantly, testing these serial transfer lineages before and after laboratory passage allowed us to distinguish the impact of the mutational load (i.e., mutations that had become fixed in the genome but no longer involved physical DNA mispairing) from ongoing mutational processes (i.e., active genomic lesions consisting of unresolved mismatches or other DNA damage) because only the former is expected to increase over the serial transfer process. Collectively, our experiments described herein support the hypothesis that random mutational damage to the genome promotes the development of competence in *S. pneumoniae*.

## Materials and Methods

### Bacterial growth conditions, strains and mutagenesis

Cultures of *S. pneumoniae* were grown in C+Y_CAA_ medium [11], [14] for routine propagation and mutagenesis whereas *E. coli* was grown in LB medium. Antibiotics were used at the following concentrations for selection: erythromycin, 2 µg/ml (*S. pneumoniae*) and 500 µg/ml (*E. coli*); streptomycin, 500 µg/ml; kanamycin, 500 µg/ml. Strains of *S. pneumoniae* and *E. coli* used in this study are listed in Table 1. Targeted mutations were generated in *S. pneumoniae* by PCR ligation mutagenesis [15] using primers listed in Table 2. Unmarked, in-frame deletions were produced in *mutX*, *comA* and *hexA* using the couterselectable Janus cassette [16]. Allelic replacement with the *aph3* kanamycin resistance cassette was used to interrupt the genes *bgaA* and *hexB*. A reporter plasmid with *lacZ* on pMU1328 was constructed as previously described [11]. A G226T mutation converting codon 76 from GAG to TAG was introduced by QuikChange mutagenesis (Agilent Technologies, Santa Clara, CA). Repair of the K56T mutation in pneumococcal *rpsL* was accomplished by transformation with a PCR product encoding wild-type *rpsL* followed by screening for streptomycin-sensitive colonies. The frequency of such colonies during screening was approximately 1 in 100.

**Table 1:** Strains used in this study.

*Streptococcus pneumoniae*		
**Strain**	**Characteristic(s)**	**Reference or source**
R895	R6 laboratory strain derivative with *ssbB’-luc* fusion	[17]
KSP86	R895, but *rpsL* K56T	[11]
OAP1	KSP86, but *mutX::*Janus	this work
OAP5	OAP1, but *mutX* ▵37-420	this work
OAP6	OAP1, but wild-type *mutX*	this work
OAP7	OAP5 with KSE2 plasmid	this work
OAP8	OAP6 with KSE2 plasmid	this work
OAP13	OAP7, but *bgaA::aph3*	this work
OAP14	OAP8, but *bgaA::aph3*	this work
DCP37	OAP5 with DCE6 plasmid	this work
DCP39	OAP6 with DCE6 plasmid	this work
APP1	DCP37, but *bgaA::aph3*	this work
APP2	DCP39, but *bgaA::aph3*	this work
MSP19	KSP86, but *hexA::*Janus	this work
MSP20	MSP19 with *hexA* ▵193-2346	this work
MSP22	MSP20, but wild-type *rpsL*	this work
KSP191	R895, but *hexB::aph3*	this work
KSP198	MSP22, but *hexB::aph3*	this work
AGP5	MSP20, but *comA::*Janus	this work
AGP6	AGP5 with *comA* ▵427-2073	this work
AGP7	AGP6, but wild-type *rpsL*	this work
Escherichia coli		
**Strain**	**Characteristic(s)**	**Reference or source**
KSE2	DH5α, but transformed with *lacZ* reporter plasmid (intact *lacZ* ORF)	[11]
DCE6	DH5α, but transformed with *lacZ* G226T reporter plasmid (TAG codon)	this work

**Table 2:** PCR primers used in this study.

Usage and primer name			Primer sequence (5′ → 3′)
Janus cassette amplification			
	JANUS765F32		GATCGGATCCGTTTGATTTTTAATGGATAATG
	JANUS2109R30		ACCTGGGCCCCTTTCCTTATGCTTTTGGAC
*aph3* amplification			
	JANUS765F32		GATCGGATCCGTTTGATTTTTAATGGATAATG
	JANUS1673R24		ACCTGGGCCCCTCTCCTGTGTTTTTTTATTTTTGG
*rpsL* region amplification			
	RPSL540F25		GCTGTAGACCATTTTTTTCTCAGTG
	RPSL1886R20		GAATGCACGGTTAGCTTCAG
*mutX* ▵37-420 overlap-extension			
	5′-region:	MUTX3151R22	CAGTAAAAATGAAGGAGTCGGC
		MUTX2430del2045	GTATCCAACAATTTATCCCCATCATTATCAATGTAACAAATCGTCGC
	3′-region:	MUTX2045del2430	GCGACGATTTGTTACATTGATAATGATGGGGATAAATTGTTGGATAC
		MUTX1234F22	GCTGTGACTTCTGCACCTAACC
*bgaA*::*aph3* mutagenesis			
	5′-region:	BGAA1408F21	GCCACTCTTCCTTCTTTCAGG
		BGAA2059RBAMHI	GATCGGATCCCCCACAGCAAACTTACGAATG
	3′-region:	BGAA8609FAPAI	ACCTGGGCCCAGCAGCAGTAGCAGCAGGTTTAGC
		BGAA9104R23	GGTAGACAGATTTCGCTCAACGC
*hexA* ▵193-2346 overlap-extension			
	5′-region:	HEXA4965R23	AAGAAATTCAAGATCGGTTGGAG
		HEXA4344del2189	CTCTCTGTTCCTTGATTCTCTAGCTGCATAGGGATCGGATTGTCGG
	3′-region:	HEXA2189del4344	CCGACAATCCGATCCCTATGCAGCTAGAGAATCAAGGAACAGAGAG
		HEXA1430F21	AACGAACCGATGAACAAGAGG
*hexB*::*aph3* mutagenesis			
	5′-region:	HEXB1897F20	AGGGATTGGGCTCCTATGTG
		HEXB2721BAMHI	ACGAGGATCCCAGGCAAGGACACAAAACCTG
	3′-region:	HEXB3236APAI	AGCAGGGCCCGAGAAAGCCTGCTAACTACGACC
		HEXB4014R22	TTTGAACACCTGATTTTATGCG
*comA* ▵427-2073 overlap-extension			
	5′-region:	COMAB1786F23	TTTTGTTTAGTGATTGGGGTAAG
		COMA2426del4074	CTTCGACAATCTTGCCCTGTGGACTAGGTGCCATAAAAAGAGTC
	3′-region:	COMA4074del2426	GACTCTTTTTATGGCACCTAGTCCACAGGGCAAGATTGTCGAAG
		COMA5281R20	TAGCGAACAGAATCACCGAC
l*acZ* G226T mutagenesis			
	LACZG226TF		GCTGGAGTGCGATCTTCCTTAGGCCGATACT
	LACZG226TR		AGTATCGGCCTAAGGAAGATCGCACTCCAGC

### Luciferase reporter assays

Activation of the competence system was determined by measuring the activity of a luciferase reporter in strains with an *ssbB*’-*luc* transcriptional fusion [17]. This reporter consists of the *ssbB* promoter and the first 44 nucleotides of the *ssbB* coding sequence followed by the luciferase gene in a different reading frame. An intact copy of *ssbB* is also retained under control of its native promoter in all strains. *ssbB* is specifically induced during competence, and activity of this reporter has been demonstrated to reflect competence for transformation [5], [17]. As described previously [11], strains were inoculated at a 1:400 dilution into 200 µl aliquots of C+Y_YB_ medium containing 0.65 mM D-luciferin in 96-well microplates. Luminescence and OD_620_ were monitored during incubation at 37°C using a Synergy2 plate reader (BioTek, Winooski, VT). For assays involving the mutagenic nucleotide analog dPTP (the triphosphate form of the nucleoside deoxyribosyl dihydropyrimido[4,5-*c*][1], [2]oxazin-7-one), this reagent was added to samples at the beginning of the growth period. When testing competence in the mutation accumulation lineages, assays were initiated by inoculating single colonies of each lineage into culture medium rather than from dilutions of starter cultures in order to eliminate additional culture steps and thereby reduce the opportunity for selection.

Activity of the luciferase reporter was normalized to the density of the culture at the time of measurement. This normalization was based on the OD_620_ for each sample during growth rather than on the actual number of CFU per ml of culture. We determined, however, that the number of CFU per ml of culture did not differ between the wild type and either the *mutX* or *hexB* deletion strains when measured at an OD_620_ near 0.15 (data not shown). A small difference between these two density measures was found for the *hexA* deletion strains and for samples treated with dPTP as compared to the wild type or untreated samples, as described in the Results.

Statistical comparisons were performed by 2-way repeated-measures ANOVA with Bonferroni’s correction using Prism 4.0 software (GraphPad Software, La Jolla, CA). Results of ANOVA testing are presented using a *P*-value as well as the *F*-statistic (*F*_[df1,df2]_) from which the *P*-value is derived with the associated degrees of freedom df1 and df2.

### Transformation assays

Samples were prepared as described for the luciferase reporter assay but with the addition of purified genomic DNA encoding the *rpsL* K526T mutation at a final concentration of 2.5 µg/ml. Microplates were incubated as above, and samples were harvested upon reaching stationary phase. The frequency of transformation to streptomycin resistance was determined by serial dilutions and quantitative colony counting on plates with and without antibiotic.

### UAG suppression assays

Suppression of a UAG codon was determined by comparing the ratio of beta-galactosidase activity in pneumococcal cultures expressing *lacZ* reporters with and without the premature stop codon. LacZ activity was measured as previously described [11]. Disruption of *bgaA*, encoding an endogenous pneumococcal galactosidase, was employed to reduce the background activity in these assays.

To screen for mutational reversion of the *lacZ*_UAG_ reporter, colonies were plated on media containing X-gal (5-bromo-4-chloro-3-indolyl-β-D-galactopyranoside) at a density of approximately 10,000 per plate and were examined for blue coloration under a stereomicroscope. At this density, colonies (less than 0.5 mm diameter each) occupied less than 40% of the surface of an 8 cm-diameter agar plate. Controls in which the isogenic strain containing the wild type *lacZ* reporter was added to the *lacZ*_UAG_ sample confirmed the ability to detect rare colonies expressing functional LacZ under these conditions.

### Serial transfer and fitness of mutation accumulation lineages

Twelve lineages each were initiated from single colonies of strains OAP5 (*mutX* deletion) or OAP6 (wild-type *mutX*). Each lineage was propagated every 12 h by serial transfer onto sectors of fresh THY agar plates (Todd-Hewitt agar supplemented with 0.2% yeast extract) of single colonies chosen at random by selecting the most well-separated colony present on an initial field of view under a stereomicroscope. Samples were preserved after growth cycles 1 and 15 by suspending patches of colonies in 1 ml of THY broth containing 16% glycerol and stored at -75°C for subsequent testing. With the exception of an increased transfer frequency (every 12 h rather than every 24 h), this procedure was the same as the serial transfer regimen we have characterized previously in which pneumococcal colonies undergo exponential phase growth for the first 10 to 12 hours of each growth cycle [18].

Experimental lineages (streptomycin-resistant) were assigned consecutive odd numbers and were separated during transfer by plate sectors inoculated with lineages of a streptomycin-sensitive control strain as a check for contamination between lineages. The expected pattern of resistance was maintained following 15 cycles of propagation. The fitness of each strain for growth on THY agar was determined by dissecting the most well-separated colony within a sector of a plate inoculated with the sample and measuring the number of CFU within the colony by dilution and colony counting [18]. The log_2_(CFU/colony) was calculated as a measure of the net growth rate of the colony. The ratio of this value for each individual isolate to the average value for the set of unpassaged lineages of the same *mutX* genotype at growth cycle 1, which were processed in parallel with each assay, was used as a proxy for fitness as described previously [18]. Fitness for growth in broth culture was measured by determining the maximum rate of change in log_10_(OD_620_) and calculating the ratio of this value for each isolate to the average value for the set of growth cycle 1 lineages measured in parallel [18].

### Whole-genome resequencing

In order to avoid bottlenecks and minimize selection during the growth of serial transfer strains for DNA purification, strains were inoculated from frozen stocks onto THY agar plates, and after approximately 10 h growth all resulting colonies were inoculated into THY broth and harvested before reaching stationary phase. Genomic DNA was then isolated using the MasterPure Gram Positive DNA purification kit (Epicentre Biotechnologies, Madison, WI). Genomic libraries with bar-coded adapters for Illumina DNA sequencing were prepared at the Tufts University Core Facility (TUCF) as described by Lazinski and Camilli (http://tucf.org/htseq_protocol_for_illumina_paired.pdf). Sequencing using single-ended reads was conducted with up to six samples multiplexed per lane. Bioinformatic analyses by the TUCF mapped the reads onto the R6 pneumococcal genome sequence. More than 2 million reads were generated for each genome (median 11,759,849 reads per sample) of which at least 96% (median 99%) were mapped to the R6 sequence and covered the entire genome with the exception of the *mutX* region deleted in the ancestral strains. The average depth of coverage for the resequenced genomes was at least 104 reads per position (median 558). In order to capture mutations that had become fixed in a lineage during serial transfer rather than minor variants arising during preparation of the DNA sample, SNPs were defined as variants present in at least 95% of the reads for a given position. Variants present in the ancestral sequence as defined by resequencing of the OAP5 genome and a representative genome from growth cycle 1 were also excluded.

## Results

### Effects of *mutX* deletion on transcriptional errors and competence

To investigate the relationship between mutational load and competence, we first analyzed the development of competence in mutator strains of *S. pneumoniae*. Pneumococcal *mutX* encodes a nucleotide triphosphate hydrolase homologous to *mutT* of *E. coli* [19]. MutT preferentially degrades oxidized guanine nucleotides that have the potential to mispair with adenine [20], [21]. Because such mispairings arise with both 8-oxo-guanosine triphosphate (8-oxo-rGTP) and 8-oxo-deoxyguanosine triphosphate (8-oxo-dGTP), this process is a source of errors during both DNA replication and transcription. MutT improves the fidelity of both these processes by cleansing the intracellular nucleotide pool [20], [21]. In *S. pneumoniae*, loss of *mutX* increases the frequency of AT to CG transversions by over 300-fold [22]. The impact of *mutX* on transcriptional fidelity, however, had not been studied.

**Figure 1:**
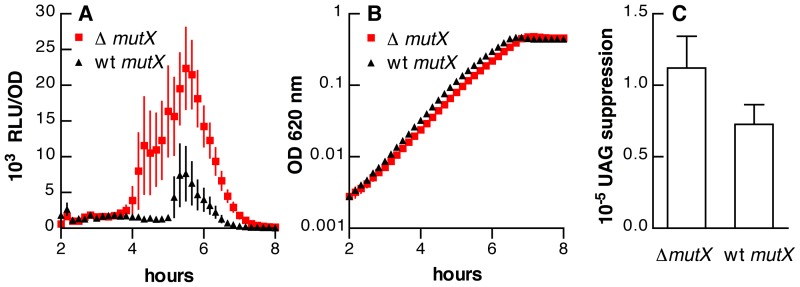
Effects of *mutX* on competence and UAG codon suppression. (A) Luciferase activity, expressed as relative light units/optical density (RLU/OD_620_), for strains OAP5 (▵*mutX*, red squares) and OAP6 (wild-type *mutX*, black triangles) containing the *ssbB*’-*luc* reporter. (B) Optical density (OD_620 nm_) for the same cultures shown in panel A. Symbols represent means ± SEM for 20 replicate cultures. (C) Suppression of a premature UAG stop codon in a *lacZ* reporter for pneumococcal strains with and without *mutX*. Bars represent means ± SEM, n = 14.

The role of *mutX* in suppressing transcriptional errors was assayed using a *lacZ* reporter in which a premature stop codon was inserted such that mispairing of 8-oxo-rGTP with adenine on the template strand during transcription would be able to regenerate the wild-type GAG codon (glu76) in place of the UAG codon (stop). The frequency of such errors was determined by comparing LacZ activity from this reporter to that of an isogenic strain with an intact *lacZ* sequence. As expected, loss of *mutX* was associated with increased suppression of the UAG codon (Figure 1C, *P* = 0.04 by paired t-test).

We considered the possibility that the increased LacZ activity of the *mutX* strain might result from mutational reversion of the reporter gene in a small subpopulation of the bacteria that were assayed. However, plating of more than 10^6^ colonies of the *lacZ*_UAG_ reporter strain on media with X-gal gave no evidence of such reversion. It should be noted that the basal frequency of transcriptional errors (median frequency 7.3×10^−6^, Figure 1C) is more than 300-fold higher than the apparent frequency of the transversion mutations that are required for reversion of the *lacZ* stop codon (median frequency less than 2.3×10^−8^ based on the frequency of spontaneous streptomycin resistance, which can arise from a similar transversion in *rpsL*). As a result, the small percentage increase (approximately 1.5-fold) resulting from loss of *mutX* in these relatively frequent transcriptional errors is anticipated to generate an absolute error burden that may be larger than that resulting from a 300-fold increase in the low basal mutational frequency in the same strain.

With regard to competence, we found that deletion of *mutX* significantly increased the expression of the *ssbB*’-*luc* reporter (Figure 1A; *F*[_1, 38_]  = 11.0, *P* = 0.0021) without a substantial effect on the growth rate (Figure 1B; *F*[_1, 38_]  = 0.077, *P* = 0.78). These findings demonstrate that pneumococcal *mutX*—in addition to its established role in preventing errors during DNA replication—reduces the frequency of transcriptional errors. Furthermore, loss of *mutX* increases the expression of the competence-specific *ssbB* promoter. These initial assays, however, did not distinguish the impact of the increased transcriptional errors and the increased mutational load that both accompany the loss of *mutX*. To test specifically the impact of mutational load on competence, we then tested other pneumococcal mutator strains, chemical mutagenesis and mutation accumulation lineages of *S. pneumoniae*.

### Loss of mismatch repair enhances competence

**Figure 2:**
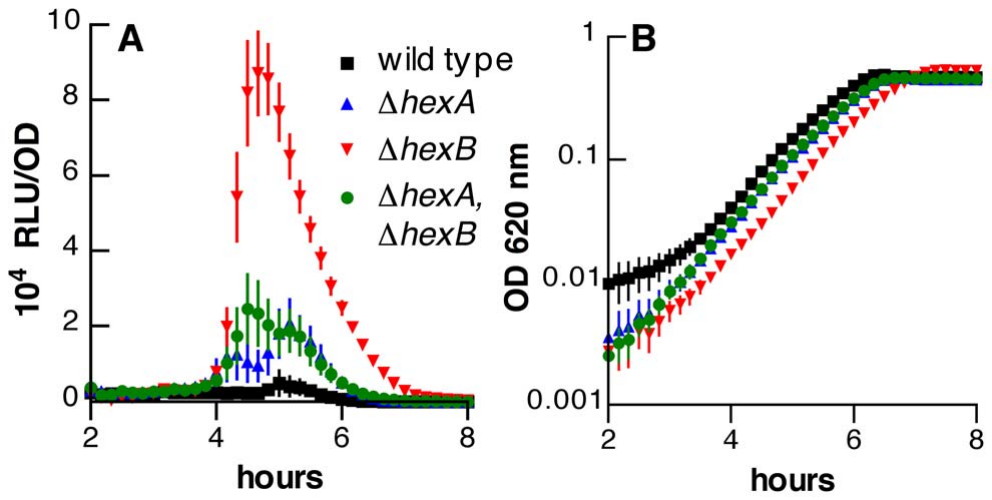
Modulation of competence signaling by the *hex* mismatch repair system. (A) Luciferase activity of strains R895 (wild type, black squares), MSP22 (▵*hexA*, blue triangles), KSP191 (▵*hexB*, red inverted triangles) and KSP198 (▵*hexA*, ▵*hexB*; green circles), all containing the *ssbB*’-*luc* reporter. Symbols represent means ± SEM for 24 replicate cultures. For MSP22 versus R895, *F*_[1, 46]_ = 9.56 and *P* = 0.0034. For KSP191 versus R895, *F*_[1, 46]_ = 140 and *P*<0.0001. For KSP198 versus R895, *F*_[1, 46]_  = 10.6 and *P* = 0.0021. (B) Optical density for the same cultures shown in panel A.

The pneumococcal mismatch repair system is encoded by the genes *hexA* and *hexB*, which are found at separate chromosomal loci homologous to *mutS* and *mutL* of *E. coli*, respectively [23], [24]. Pneumococcal *hex* mutants display mutator phenotypes with 10- to 20-fold increases in the mutation rate [25]. As expected, *hex* mutants display higher transformation frequencies for markers that would otherwise be efficiently recognized by mismatch repair following recombination [26]. The impact of the *hex* system on activation of competence signaling, however, has not been studied. We found that deletion of either *hexA* or *hexB* promoted development of competence, as measured by an *ssbB*’-*luc* reporter fusion (Figure 2). Interestingly, the impact of *hexB* deletion was significantly stronger than that of either *hexA* deletion or a combination of the two mutations.

Because activity of the luciferase reporter was normalized to concurrent OD readings during the assay, we tested the relationship between bacterial density and OD_620_ for these strains as had been done for the *mutX* deletion. The *hexB* deletion strain (KSP191) did not show a significant change in number of CFU/ml per OD unit as compared to the wild type (R895, data not shown). For strains with the isolated *hexA* deletion (MSP22) and the dual *hexA hexB* deletions (KSP198), however, this value was 60±10% and 55±13% that of the wild type, respectively. This observation implies that the actual difference between these strains and the wild type in terms of luciferase activity per cell may be somewhat higher than suggested by the values shown in Figure 2A as a result of more wild-type than mutant bacteria at a given OD. The difference in CFU/ml, however, is not sufficient to explain the approximately 4-fold greater induction of the competence reporter in the strain with the isolated *hexB* deletion as compared to either strain lacking *hexA*.

### Chemical mutagenesis

**Figure 3:**
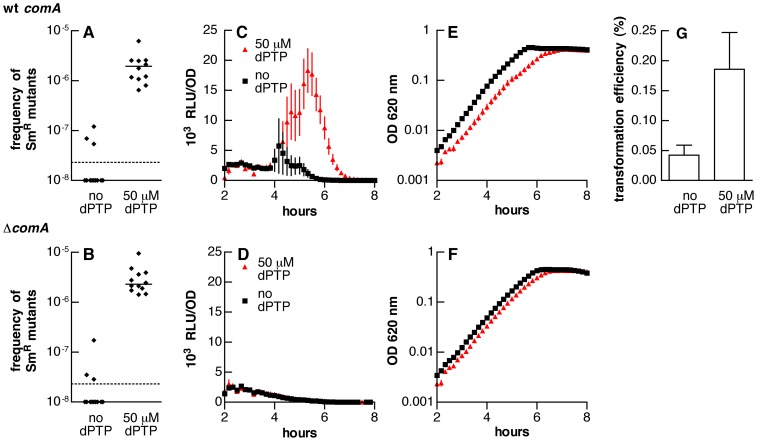
Impact of dPTP on mutagenesis and competence development. (A, B) Frequency of streptomycin-resistant (Sm^R^) colonies in cultures of (A) MSP22 (▵*hexA*) and (B) AGP7 (&▵;*comA,* ▵*hexA*) grown in the presence or absence of 50 µM dPTP. The limit of detection of the assay is shown with a dotted line. Samples without resistant colonies are plotted at the bottom of the graph. Solid lines represent median values. *P*<0.0001 for both strains when comparing mutation frequencies with and without dPTP by Mann-Whitney *U* tests. (C, D) Luciferase activity of cultures grown with 50 µM dPTP (red triangles) or without additive (black squares) for (C) MSP22 and (D) AGP7. Both strains contain the *ssbB*’-*luc* reporter. Because loss of *hexA* also promotes competence, the medium for all these assays was adjusted to pH of approximately 7.15 to reduce development of competence in untreated cultures. Symbols represent means ± SEM for 16 replicate cultures. (E, F) Optical density for the same cultures shown in panels C and D, respectively. (G) Frequency of transformation yielding streptomycin resistance in cultures treated with or without 50 µM dPTP and exposed to genomic DNA encoding the *rpsL* K56T mutation. Bars represent means ± SEM, n = 16. *P* = 0.04 by unpaired t-test. Control samples without addition of DNA consistently had frequencies of streptomycin resistance of less than 1 in 10^7^ colonies (no dPTP) or near 1 in 10^6^ colonies (with dPTP).

To introduce random coding errors in the genome while limiting damage to DNA and other cellular macromolecules we selected mutagenesis with the nucleotide analog dPTP. This cytidine nucleotide analog forms base pairs with either adenine or guanine nucleotides resulting in both GC to AT and AT to GC transitions [27]. Addition of 50 µM dPTP to pneumococcal culture medium increased the frequency of spontaneous streptomycin-resistant mutants from a basal level of less than 2.3×10^−8^ to a frequency of 2.0×10^-6^ in the setting of the *hexA* deletion (Figure 3A; strain MSP22, *P*<0.0001) with defective mismatch repair. Consistent with our findings for the mutator strains, this concentration of dPTP also promoted the expression of competence (Figure 3C; *F*_[1, 30]_  = 16.9, *P* = 0.0003). We observed, furthermore, that dPTP induced expression of the competence reporter in a wild-type background with intact mismatch repair, albeit less strongly (Supplementary Figure S1). Because dPTP reduced the number of CFU/ml in treated cultures to 76±8% of that seen in the absence of treatment, the induction of luciferase activity per cell resulting from dPTP may be slightly greater than evident from the values normalized to OD shown in Figures 3C and S1 (as discussed for the case of *hexA*).

Induction of the *ssbB*’-*luc* reporter has been demonstrated to occur specifically during the pneumococcal competence response [5], [17]. This specificity is the result of the presence of a distinctive sequence known as the Com box in the promoter of *ssbB* and other late stage competence genes that is recognized by the alternative sigma factor ComX but not the housekeeping sigma factor σ^A^ [28]. Notably, ComX is the only alternative sigma factor encoded by the genome of *S. pneumoniae* [29] and is a central regulator of competence. Nonetheless, in order to confirm that increased expression of the *ssbB*’-*luc* reporter corresponded to the development of competence, we tested the ability of cultures treated with dPTP to undergo transformation. As expected, these assays demonstrated that 50 µM dPTP increased the frequency of transformation in treated samples (Figure 3G). The experimental conditions for our assays were selected to be near the threshold of competence development in order to detect the impact of small mutational perturbations as discussed further below, and accordingly not all samples treated with dPTP expressed competence. Conversely, several samples among the group without dPTP displayed evidence of transformation. Although the average frequency of transformation among the 16 samples treated with dPTP was only 0.19% (Figure 3G), transformation frequencies up to 0.87% were observed for the samples in which competence was induced. These values are in the range reported for transformation of *S. pneumoniae* under more favorable conditions [5].

To test whether the induction of competence by dPTP required the established peptide pheromone signaling pathway, we evaluated the impact of the mutagen in the context of a *comA* deletion, which prevents the export of CSP [9]. In this background, dPTP was unable to stimulate competence (Figure 3D) despite causing a similar mutagenic effect, as indicated by a frequency of streptomycin-resistant mutants of 2.3×10^-6^ (Figure 3B; strain AGP7, *P*<0.0001).

### Mutation accumulation experiments

To separate the effects of DNA replication errors from the effects of transcriptional errors in the *mutX* background, we conducted a mutation accumulation experiment in which independent lineages of the mutator strain were passaged under conditions of relaxed selection. By comparing isolates at the end of this process to those at the beginning, we were able to examine the impact on competence of secondary mutations that accumulated in this setting. Because the *mutX* deletion was present in these strains before and after passage, differences in the transcriptional error rate related to *mutX* could be excluded as a factor in these experiments. These assays also served to distinguish the effects of an established mutational load from the effects of ongoing mutagenic processes as outlined in the Introduction.

Starting with a single colony of the *mutX* deletion strain (OAP5), twelve independent lineages were initiated from separate daughter colonies and propagated by serial transfer to fresh agar plates every 12 hours. The same serial transfer process was carried out in parallel with 12 control lineages derived from a single colony of an isogenic strain in which *mutX* was intact (OAP6). For each transfer step, a single colony was chosen at random to be spread on the next plate. Such repeated single colony bottlenecks have been a valuable tool in bacterial population genetics for examining random mutational processes while severely limiting the potential for adaptation by imposing strong genetic drift at low effective population sizes [30], [31]. We had, however, recently conducted another mutation accumulation experiment in *S. pneumoniae* in which we observed unanticipated adaptation associated with the second half of the daily colony growth cycle during which period bacterial colonies were in stationary phase [18]. Consequently, the current experiment was conducted by propagating colonies every 12 hours rather than every 24 hours to eliminate effectively the opportunity for such selection during stationary phase.

The fitness of initial and derived strains was evaluated both in the context of colonies growing on agar plates and by measuring the maximum growth rates of isolates in broth culture. In neither setting was evidence seen for adaptation during the serial transfer process (Table 3). Although one isolate (lineage 5) in the *mutX* deletion group showed a trend toward loss of fitness in both assays—as would eventually be expected in a prolonged mutation accumulation experiment due to the random fixation of mildly deleterious mutations—this change was not significant when analyzed by ANOVA corrected for multiple comparisons. The observation that fitness did not rise during laboratory passage provides evidence that the serial transfer process used here did not permit adaptation among the lineages.

**Table 3:** Fitness of mutation accumulation lineages after 15 growth cycles.

▵*mutX* lineages			wild-type *mutX* lineages		
Lineage number	Colony growth^a^	Broth growth^a^	Lineage number	Colony growth^a^	Broth growth^a^
1	1.00±0.06	1.06±0.09	25	0.98±0.06	1.00±0.05
3	0.97±0.07	1.01±0.05	27	0.99±0.08	1.01±0.04
5	0.94±0.05	0.92±0.01	29	1.00±0.06	0.98±0.04
7	1.02±0.05	0.97±0.04	31	0.99±0.04	0.98±0.04
9	1.01±0.06	1.05±0.02	33	1.02±0.05	1.01±0.06
11	0.94±0.09	1.01±0.08	35	1.02±0.03	0.95±0.06
13	1.00±0.03	1.02±0.03	37	1.01±0.07	0.97±0.03
15	0.94±0.07	1.02±0.04	39	1.01±0.02	0.99±0.03
17	1.02±0.02	0.97±0.09	41	1.04±0.05	1.02±0.06
19	1.01±0.04	1.00±0.11	43	1.01±0.07	0.94±0.07
21	0.97±0.11	1.03±0.04	45	0.99±0.07	0.99±0.02
23	1.01±0.03	1.02±0.06	47	0.97±0.04	1.02±0.03
**average** b	**0.99±0.03**	**1.01±0.04**	**average** b	**1.00±0.02**	**0.99±0.02**

a Mean fitness ± SD, n≥5 for each lineage.

b Mean fitness for set of lineages ± SD, n = 12 lineages.

**Figure 4:**
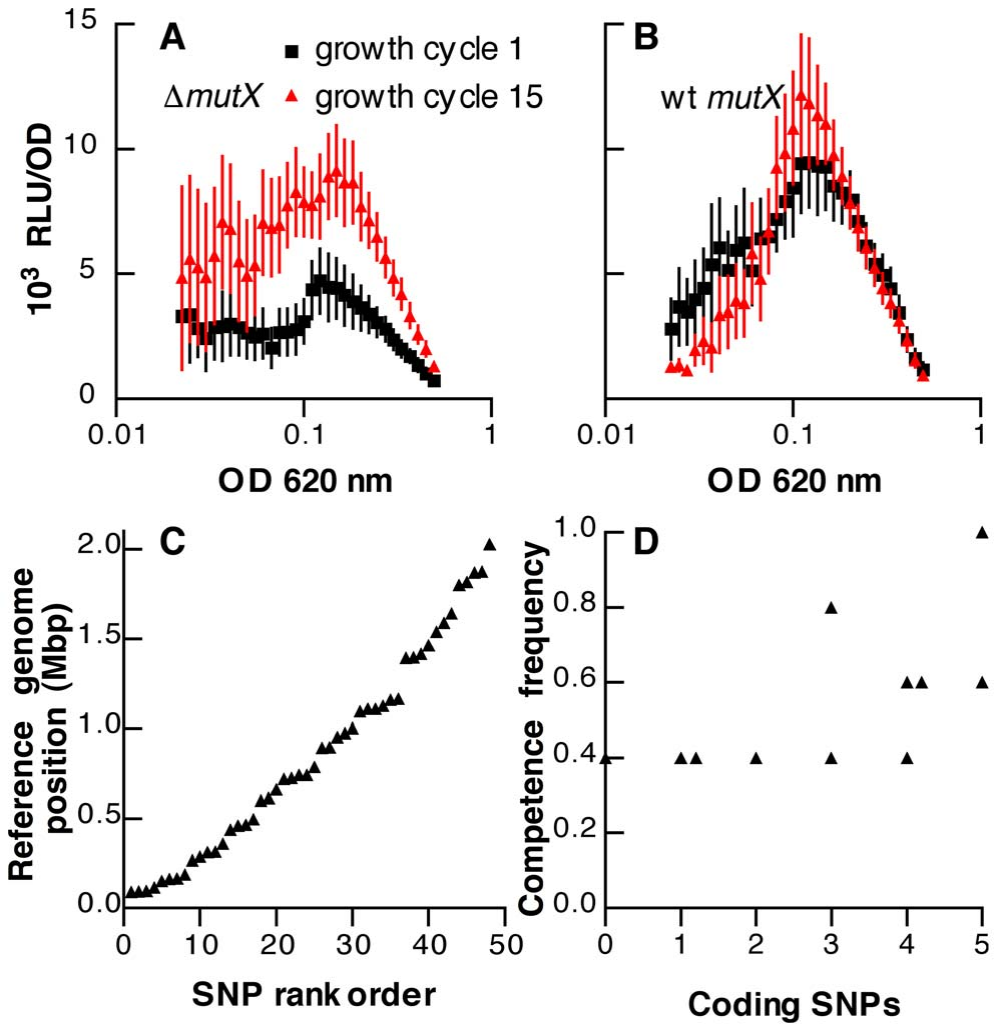
Effect of mutation accumulation on pneumococcal competence. (A, B) Activity of an *ssbB*’-*luc* competence reporter in (A) 12 lineages of strain OAP5 (▵*mutX*) and (B) 12 lineages of strain OAP6 (wild-type *mutX*) before (black squares) and after (red triangles) 15 growth cycles of serial transfer. Symbols represent means ± SEM for 12 independent lineages. Because assays were started by inoculation of individual colonies into each sample well rather than using standardized starter cultures, growth times of samples were variable. Results are therefore displayed by plotting luciferase activity against optical density of the culture. (C) Distribution of 48 SNPs identified during resequencing of 11 lineages in the &▵;*mutX* background and ordered by location in the R6 reference genome. (D) Frequency of competence development among 11 sequenced &▵;*mutX* lineages as compared to the number of nonsynonymous SNPs identified in coding regions of each genome. Competence frequency was determined as the proportion of replicate samples for a given isolate that displayed luciferase activity above a baseline level during the assay. This baseline was defined by the set of samples that did not develop a burst of luminescence at any point but rather showed consistently low activity levels (approximately 1000 RLU/OD_620_ at the start of exponential phase) that slowly declined further during growth.

Competence assays were then conducted to examine for changes in behavior of the lineages following propagation. Among the set of 12 lineages in the *mutX* deletion background, a significant increase in expression of competence was seen for the isolates at growth cycle 15 as compared to the same set of lineages at growth cycle 1 (Figure 4A; *F*_[1, 22]_  = 4.65, *P* = 0.042). For the wild-type lineages, however, no change in competence was associated with the serial transfer process (Figure 4B; *F*_[1, 22]_  = 0.032, *P* = 0.86). The graphs in Figures 4A and 4B represent the mean behaviors of the two sets of serial transfer lineages with regard to competence. Because each lineage was expected to accumulate different mutations at random during the serial transfer process, however, it was anticipated that the induction of competence would be variable across the *mutX* deletion lineages. Such variability was evident when the lineages were analyzed individually and is shown in Supplementary Figure S2. This experiment had been designed to determine only whether serial transfer and mutation accumulation would result in an increase in competence on average among the set of passaged lineages as a whole rather than to characterize precisely the impact of such passage on competence for any individual lineage. Substantial additional experimental replicates of the competence assay for each lineage would have been required to achieve the latter objective but were precluded by the exhaustion of the low density frozen stocks as discussed below. Given the limited statistical power available when lineages were examined individually, only for lineage 5 was the serial transfer process shown to significantly increase competence (Figure S2; *F*_[1, 8]_  = 38.1, *P* = 0.0003). The impact of serial transfer on competence, however, did not appear to be limited to lineage 5 because excluding this lineage from the overall analysis of the group of 11 other lineages did not substantially affect the results of the ANOVA (*F*_[1, 20]_  = 4.04, *P* = 0.058 without lineage 5 as compared to *F*_[1, 22]_  = 4.65, *P* = 0.042 with lineage 5).

In designing these assays, the culture conditions were adjusted such that 25–50% of the growth cycle 1 cultures developed spontaneous competence for both sets of lineages. This consideration was necessary in order to permit the experiment to detect changes that might either promote or repress the expression of competence. For this adjustment, we took advantage of the previous observation that the frequency with which a pneumococcal culture develops spontaneous competence is sensitively dependent on the pH of the culture medium over a range from pH 7.2 to 7.4 [11]. Because deletion of *mutX* increases the development of competence at baseline (Figure 1A), the medium used for the *mutX* deletion lineages (Figure 4A) was therefore less permissive for competence than that used for the wild-type lineages (Figure 4B). Collectively, these results from serial transfer support the hypothesis that the accumulation of secondary mutations in the *mutX*-deficient background increases the expression of competence independently of the effect *mutX* on transcriptional fidelity.

### Resequencing of mutation accumulation lineages

Secondary mutations that had developed in the *mutX* deletion lineages during the serial transfer process were identified by genome resequencing. Eleven of the twelve original isolates from growth cycle 15 were available for sequencing. The frozen stock for one isolate (lineage 3) had become exhausted during the process of conducting competence and fitness assays, and attempts to grow this sample for sequencing were repeatedly unsuccessful. Additional stocks subsequently became exhausted but were sequenced successfully. It should be noted that the density of bacteria within stocks of the mutation accumulation strains was particularly low because these stocks were prepared by directly harvesting only the limited number of bacteria that had grown 12 hours after seeding a plate with a single colony. Samples were not amplified by growth in broth before preservation in order to avoid the opportunity for selection that this step would have provided.

Between 1 and 9 single-nucleotide polymorphisms (SNPs) that were not found in the ancestral sequence were identified in each of the passaged lineages (Supplementary Table S1). These SNPs were detected in 96.6–100% (median 99.8%) of the 57-1265 reads (median 548) covering that position for a particular isolate. In contrast, no deletion or insertion polymorphisms were identified to have reached fixation during serial transfer. Of the SNPs, 56% represented the unilateral AT to GC transversions that are expected to result from enhanced mutagenesis due to 8-oxo-dGTP in the absence of *mutX* [22]. The mutations were distributed broadly across the genome (Figure 4C) rather than being clustered at a small number of loci and affected genes representing a wide range of different pathways. We did identify, however, one isolate in which two separate SNPs had occurred in a single gene (the pneumococcal histidine triad protein *phtE*). Two separate isolates were also found to share an identical SNP in the gene *fhs*, encoding formate-tetrahydrofolate ligase, and three isolates had mutations in different heat shock proteins. Overall, however, the evidence for convergent evolution among the lineages at the sequence level was minimal, consistent with the lack of adaptation seen in fitness assays. Importantly, no known regulators of competence were among the genes in which SNPs were identified. In particular, the two-component system for which a mutation was observed in the histidine kinase (HK04) for one lineage does not appear to regulate competence based on microarray analysis of gene expression following deletion of the cognate response regulator [32].

Analysis of the frequencies of nonsynonymous and synonymous mutations also revealed no evidence of positive selection. Of the 48 SNPs that were found in the passaged *mutX* deletion lineages, 44 occurred in predicted coding sequences, and 32 of these resulted in nonsynonymous substitutions (Supplementary Table S1). The ratio (dN/dS) of the frequency of nonsynonymous substitutions to that of synonymous substitutions suggested a small underrepresentation of nonsynonymous changes for both AT to GC transversions (dN/dS = 0.65) and for transitions (dN/dS = 0.78). These values argue against positive selection for new adaptive traits but would be consistent with a small degree of purifying selection such as expected from the elimination of lethal mutations. Due to the small total number of mutations observed, however, the dN/dS ratios were not significantly different from the value of 1 expected in the absence of selection (*P* = 0.49 for AT to GC transversions and *P* = 0.83 for transitions).

Examining the impact of mutational burden on competence, we found that the probability of an isolate developing competence at the end of the serial transfer process correlated positively with the number of nonsynonymous mutations it had acquired within predicted coding regions (Figure 4D; Spearman correlation coefficient r_S_ = 0.70, *P* = 0.02). This correlation remained significant when lineage 23 containing a SNP in histidine kinase HK04 was omitted from the analysis (r_S_ = 0.81, *P* = 0.006) or when the lineages with heat shock mutations were omitted (r_S_ = 0.81, *P* = 0.02). Overall, both our fitness data and sequencing results indicate that the serial transfer process was characterized by random accumulation of a diverse set of mutations rather than specific adaptive or regulatory changes. We therefore conclude that the development of competence in *S. pneumoniae* may be promoted by small increases in the overall mutational load of the organism.

## Discussion

Evidence has recently been developing that genetic competence in *S. pneumoniae* represents a type of bacterial stress response [10], [11]. Our studies using mutator strains, chemical mutagenesis and mutation accumulation lineages now support the conclusion that competence is stimulated in response to mutational damage in the bacterial genome. This response, however, differs from other genome stress responses such as the SOS pathway in that the inciting stimulus appears to be informational damage to the coding properties of the genome rather than physical alterations that deform the structure of the DNA such as mispaired bases or single-stranded DNA. Although these findings indicate that competence is regulated in a manner that is considerably more complex than a simple quorum-sensing system, the induction of competence by genetic damage still requires activity of the core peptide pheromone signaling circuit.

The evidence here that pneumococcal competence is stimulated by increases in the mutational load lends support to the idea that a principal function of competence is the repair of genetic damage. This damage may take the form of the gradual accumulation of mutations that individually are only mildly deleterious and therefore are difficult to remove from the population by selection. The progressive fixation of such mutations through genetic drift in small populations despite selection has been classically described as Muller’s ratchet [33]. This process may be especially problematic for an organism such as *S. pneumoniae* in which frequent transmission between hosts imposes recurrent population bottlenecks. Although the size of this transmission bottleneck has not been measured, it is almost certain to be small considering that most spread occurs by means of respiratory secretions from asymptomatic hosts [34]. Moreover, such bottlenecks are a frequent feature in the evolution of pneumococcal populations because individual hosts remain colonized with a particular pneumococcal strain for only a few weeks to months before clearing the organism [35]. These factors favor the operation of Muller’s ratchet and create a situation in which the repair of deleterious mutations through transformation may provide a selective advantage. Notably, episodes of concurrent colonization with multiple pneumococcal strains in a single host are not uncommon [36] and may represent opportunities for genetic repair because different strains are unlikely to have randomly suffered the same deleterious mutations.

It should be noted that this proposed function of competence would not prevent the action of Muller’s ratchet entirely. Rather, mutations would continue to accumulate even if there were a continuous supply of high-quality DNA available for transformation until a threshold level of mutational damage promoted the expression of competence. Furthermore, once competence developed, repair would be accomplished only by non-directed recombination and would require selection to amplify rare bacteria that had undergone a beneficial recombination event. In this scenario, the role of competence would be in reconstituting a subpopulation of organisms that has a reduced mutational load and that may have previously been lost through the action of Muller’s ratchet.

At a molecular level, our recent work on the induction of competence by translational errors may provide insight into the mechanisms responsible for the sensitivity of competence to generic coding errors. The pneumococcal protease HtrA, which is expressed on the surface of the bacterium [37], has been found to function as a conditional repressor of competence that selectively reduces the expression of competence when the frequency of errors during protein biosynthesis is low [11]. As with other members of the HtrA family such as DegP in *E. coli* [38], this protease degrades misfolded proteins by digesting at sites of exposed hydrophobic residues. Pneumococcal HtrA, however, also digests the peptide pheromone CSP in a manner that competes with its digestion of misfolded proteins [12]. In this way, protein misfolding appears to act as an indirect stimulus for competence by permitting the accumulation rather than degradation of CSP. Notably, HtrA has been shown to co-localize at least approximately with the Sec apparatus [39], suggesting that it may preferentially monitor the folding of proteins that have recently been synthesized. Mutational damage and translational errors, however, should give rise to misfolded proteins both within the cell and in the extracellular compartment. Because HtrA is expected to interact only with proteins on the surface, we anticipate that an analogous mechanism is likely to exist for monitoring errors in cytoplasmic proteins. Proteases in the cytoplasmic Clp family, which both degrade misfolded proteins [40] and repress competence through the digestion of the ComX alternative sigma factor [41], are candidates that may serve this function. This predicted multiplicity in the pathways leading from errors in protein synthesis to induction of competence, however, complicates attempts to test directly the involvement of HtrA or Clp proteases in the response to mutational damage.

We considered also the possibility that the induction of *ssbB* expression we observed under these mutation-promoting conditions might represent a generalized stress response characterized by global shifts in transcription rates rather than a competence-specific phenomenon. We have not measured transcript levels for unrelated, constitutive promoters that interact with the housekeeping sigma factor σ^A^ rather than with the competence-specific sigma-factor ComX. Although our experiments do not address the mechanism behind the increase in *ssbB* expression and cannot exclude a more general effect of genomic stress on transcription beyond the ComX regulon, the response that we have shown nonetheless appears to represent true induction of the competence system as it is accompanied by genetic transformation (Figure 3G). The magnitude of the increase in *ssbB* expression (7- to 35-fold over the pre-induction baseline in the different mutants tested and in dPTP-treated samples as well as 3- to 17- fold over the maximal levels seen in the wild-type or untreated samples) causes us to favor a mechanism involving specific activation of the competence promoters rather than a global shift in transcription rates. Whether global or specific, however, the pneumococcal response to these mutation-promoting stimuli appears to include activation of the competence-signaling pathway and is likely to have consequences for evolution of the bacterial genome.

Genome resequencing to identify SNPs in our *mutX*-deficient mutation accumulation lineages provided insight into the nature of the mutational load responsible for promoting competence under our experimental conditions. In general, the mutational load in the passaged lineages was small, consisting of 1 to 9 SNPs per strain, and did not significantly impact the fitness of the bacterium under in vitro conditions. It is possible, however, that the fitness costs of these mutations may be higher when interacting with a host during colonization. The Illumina platform that we used for genome resequencing generates short reads (40 to 100 nucleotides) that may have made the detection of larger genomic rearrangements such as duplications difficult. This limitation, however, seems unlikely to impact our conclusions substantially because the serial transfer experiment described here employed a mutator strain deficient in *mutX*, which results specifically in AT to CG transversions that are detected efficiently by this sequencing method. Notably, a set of control lineages with a wild-type *mutX* allele that were propagated in parallel did not show enhancement of competence. Furthermore, sequencing of our lineages identified only SNPs and did not reveal any deletion or insertion polymorphisms or small duplications, all of which may be detected by this method when shorter than the read length. These considerations suggest that SNPs arising from the loss of *mutX*, rather than other genomic rearrangements, were responsible for the increase in competence expression.

These experiments raise questions regarding the degree of mutational loading that may be sufficient to trigger competence in natural populations of *S. pneumoniae*. Our assays were conducted under conditions chosen to be near the threshold of competence induction even in the absence of an additional mutational burden. This choice was intended to allow detection of small changes in the propensity of a strain to enter into competence as a result of small changes in the mutational load. Assays involving more substantial genetic damage would likely have been subject to additional complications from an increased general stress response. We sought therefore to establish whether the signaling system that controls pneumococcal competence displays sensitivity to changes in the mutational burden by examining the effects of small amounts of genetic damage. Our results provide preliminary evidence for such a relationship. What degree of damage is necessary to generate a robust competence response under natural conditions remains a subject for investigation. It should be noted also that the R6 strain employed for these experiments and for many studies of pneumococcal competence was the result of extensive laboratory propagation that generated a series of strains with increasing competence for transformation (summarized in [42]). Recent sequencing of R6 and its clinical progenitor strain D39 revealed an unexpectedly high number of 81 mutational differences in R6 [42]. In light of our current observations, it is intriguing to speculate that the increased competence of R6 might result in part from its high mutational load. In this way, R6 may already be close to the mutational threshold required for induction of competence through preexisting damage.

Although our data to not allow determination of the impact of mutational load on competence in most of the serial transfer lineages when analyzed individually (except lineage 5, which has 5 coding SNPs), increased competence expression on an overall basis appeared to be primarily found among some but not all of the lineages that had 3 or more coding SNPs (Figure 4D). Demonstrating a causal relationship between such SNPs and increased competence development would ideally involve reversal of the effect on competence by genetic correction of the accumulated SNPs. Correcting these multiple, unmarked SNPs without incurring additional mutations, however, presents several challenges. Targeted mutagenesis in *S. pneumoniae* generally requires insertion and subsequent removal of a counterselectable cassette into the locus of interest. This method is impractical for mutations within essential genes such as the situation with some of the random SNPs that have accumulated in our serial transfer lineages. More importantly, the process of in vitro transformation and selection of desired clones requires growing the bacterium to a large population density that presents a heightened opportunity for new mutations to arise and to become either randomly fixed or inadvertently selected under conditions of enhanced selective pressure. In contrast, our serial transfer conditions were carefully designed to minimize selective pressures by keeping population sizes low and imposing frequent single-colony bottlenecks. We therefore anticipate that attempts to correct multiple existing SNPs would likely be confounded by the development of new mutations elsewhere in the genome. Finally, even though we have used a strategy of direct transformation with PCR products encoding SNPs to generate mutations in the essential gene *rpsL*, this method is not easily generalized to other genes for which the intended mutations do not yield a selectable phenotype such as the changes in streptomycin susceptibility associated with *rpsL*. For these reasons, conclusively establishing the relationship between the burden of coding SNPs and competence induction will need to await further investigation.

The overall distribution of mutations among the sequenced lineages was consistent with a random rather than adaptive process and supported the model that non-specific increases in the burden of mutations across the genome promoted competence. In one instance, however, the same SNP was found in two independent lineages in the gene *fhs* (encoding formate-tetrahydrofolate ligase). The strains with this mutation did not appear to have a fitness advantage to suggest convergent adaptation. Rather, we suspect that this mutation may have been already present as a minor variant within the colony that served as a founder for our 12 lineages. Another lineage demonstrated the presence of 2 separate SNPs within a single gene. This non-essential gene, *phtE*, encodes a pneumococcal histidine triad protein that has been implicated in metal ion (Zn^+2^ and/or Mn^+2^) homeostasis [43]. As with the *fhs* mutations, the strain with the *phtE* SNPs did not show a significant gain in fitness. It is possible, however, that changes in metal ion concentrations could be particularly important in the context of the enhanced susceptibility of the *mutX* deletion strain to oxidative DNA damage. In this situation, an initial mutation in *phtE* may have created a strong selective pressure favoring a second SNP balancing the effects of the first change. Alternatively, the presence of two SNPs in *phtE* may represent either a pair of random events or the occurrence of a single, earlier lesion in that region of the chromosome that was subsequently repaired through an error-prone mechanism giving rise to multiple SNPs.

While evaluating the impact of the *hex* mismatch repair system on competence signaling, we made the initially surprising observation that deletion of *hexB* induced competence more strongly than deletion of *hexA*. In considering this result, it is important to note that—despite the similarity in nomenclature—approximately 0.3 Mbp separate the loci encoding *hexA* and *hexB*. Loss of either gene, however, is sufficient to fully inactivate mismatch repair [23], [24]. Moreover, deletion of both *hexA* and *hexB* together restored a lower level of competence induction similar to that of the *hexA* mutant. These findings suggest that the enhanced competence of the *hexB* mutant might be due to effects exerted by HexA specifically when HexB is absent. As a homologue of MutS, HexA is predicted to recognize base pair mismatches and to bind DNA to form a stable clamp at such sites [44]. Subsequent repair of these mismatches, however, depends on the function of the MutL homologue HexB, without which unrepaired sites may be subject to prolonged binding by HexA. The induction of competence in this situation of HexA acting in the absence of HexB may resemble the induction of competence [5] by both the DNA crosslinking agent mitomycin C and fluoroquinolone antibiotics, which trap DNA gyrase in a cleaved complex bound to DNA [45]. We speculate that all of these processes may block the progress of RNA polymerase and thereby lead to truncated transcripts and incomplete proteins that cannot fold properly. Such unfolded proteins would then serve as a stimulus for competence in a similar manner as those resulting from SNPs or translational errors.

In characterizing the effects of *mutX* on transcriptional fidelity, we noted that the frequency of errors measured in our *lacZ* system for the wild-type strain (7.1×10^-6^) was similar to the transcriptional error rate for RNA polymerase in *E. coli* (5.0×10^-6^) [46]. However, the 1.5-fold increase in transcriptional errors that was caused by deletion of pneumococcal *mutX* was relatively small compared to the 30-fold increase that had been reported in *E. coli* with loss of the homologous gene *mutT* [20]. In contrast, the increase in mutation frequency from loss of *mutX* (over 300-fold) [22] is roughly similar to the 50- to 200-fold increase in mutation frequency seen with inactivation of MutT [21]. This discrepancy raises the possibility that MutX may be relatively more selective as compared to MutT for hydrolysis of 8-oxo-dGTP rather than 8-oxo-rGTP. Alternatively, pneumococcus may possess a second mechanism for limiting transcriptional errors due to 8-oxo-rGTP that is partially redundant with the function of MutX.

The capacity of *S. pneumoniae* to activate competence as a response to non-specific genetic damage would have important implications for the population biology of this organism. Analysis of multilocus sequence typing (MLST) databases has already provided evidence for a high frequency of recombination in natural populations of *S. pneumoniae*, in which individual nucleotide sites are 50 times more likely to change through recombination rather than mutation [47]. Even this figure may underestimate the overall frequency of pneumococcal recombination because transformation events in which the donor sequence is identical to that of the recipient are not considered. Our data suggest that pneumococci may continue to undergo frequent transformation so long as the mutational burden remains above a threshold required for competence induction. Such repeated episodes of transformation may then represent a form of non-directed genome repair in which random segments of DNA (averaging 2.3 kb per transformation but exponentially distributed in length [48]) are exchanged in a process that continues until the mutational load has been reduced. From this perspective, the pneumococcal competence system may provide a form of indirect selection limiting the accumulation of deleterious mutations. Because pneumococcal transformation has been responsible for the generation of antibiotic resistance [49] and for capsular serotype changes that reduce the efficacy of vaccination [50], such an evolutionary strategy of frequent recombination for genome repair may also underlie some of the leading clinical challenges posed by this pathogen.

## Supporting Information

Figure S1 **Impact of dPTP on competence in &▵;** ***hexA*** **and wild-type backgrounds.** Activity of an *ssbB*’-*luc* competence reporter in cultures of (A) strain MSP22 (&▵;*hexA*) and (B) strain R895 (wild-type *hexA*) grown with 50 µM dPTP (red triangles) or without additive (black squares). (C, D) Optical densities for the same cultures as in panels A and B are shown in C and D, respectively. Symbols represent means ± SEM for 31-32 replicate cultures. For the effect of dPTP on competence, *F*_[1,61]_  = 76.3, *P*<0.0001 (strain MSP22) and *F*_[1,62]_ = 15.3, *P* = 0.0002 (strain R895).
(TIFF)

Figure S2 **Effects of mutation accumulation on competence of individual serial transfer lineages.** Activity of an *ssbB*’-*luc* competence reporter in 12 independent pneumococcal lineages derived from strain OAP5 (&▵;*mutX*) and assayed before (black squares) and after (red triangles) 15 growth cycles of serial transfer. Symbols represent means ± SEM for 5 replicate cultures of most lineages. For lineage 1 at growth cycle 1 and lineage 3 at growth cycle 15, frozen stocks became exhausted during testing, and assays were limited to 3 and 4 replicates, respectively.
(TIFF)

Table S1 **SNPs found in passaged *mutx* lineages.**
(DOC)
