# Supplementary figures and images for: Competence in Streptococcus pneumoniae Is a Response to an Increasing Mutational Burden
Source: PLoS One. 2013 Aug 13;8(8):e72613. doi: 10.1371/journal.pone.0072613 (PMC3742669; doi:10.1371/journal.pone.0072613)

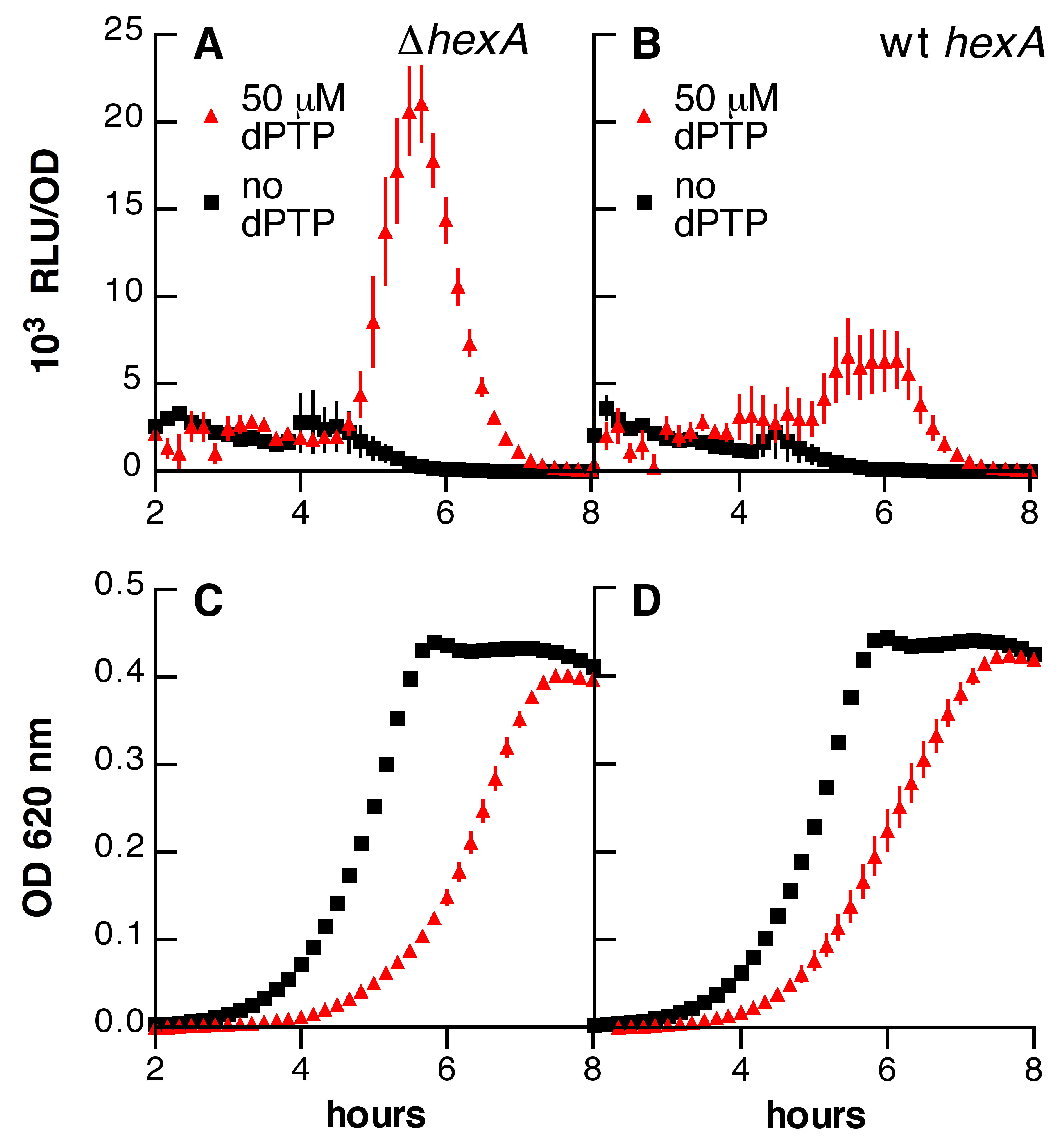

Supplement: Figure S1 — Impact of dPTP on competence in &▵; hexA and wild-type backgrounds. Activity of an ssbB’-luc competence reporter in cultures of (A) strain MSP22 (&▵;hexA) and (B) strain R895 (wild-type hexA) grown with 50 µM dPTP (red triangles) or without additive (black squares). (C, D) Optical densities for the same cultures as in panels A and B are shown in C and D, respectively. Symbols represent means ± SEM for 31-32 replicate cultures. For the effect of dPTP on competence, F [1,61] = 76.3, P<0.0001 (strain MSP22) and F [1,62] = 15.3, P = 0.0002 (strain R895). (TIFF) [file pone.0072613.s001.tiff]
