# Supplementary material for: Competence in Streptococcus pneumoniae Is a Response to an Increasing Mutational Burden
Source: PLoS One. 2013 Aug 13;8(8):e72613. doi: 10.1371/journal.pone.0072613 (PMC3742669; doi:10.1371/journal.pone.0072613)
Supplement: Table S1 — SNPs found in passaged mutx lineages. (DOC) [file pone.0072613.s003.doc]

**TABLE S1. SNPs found in passaged Δ*mutX* lineages.**

| **Position** | **Change***^a^* | **R6 gene** | **Codon change** | **Gene name** | **Annotation** |
| --- | --- | --- | --- | --- | --- |
| **Lineage 1** | | | | | |
| 164643 | **T to G** | spr0156 | Met97Arg |  | Conserved hypothetical protein, similar to NrdI family |
| 744758 | C to T | spr0748 |  |  | ABC transporter, ATP-binding protein |
| 895288 | **A to C** | spr0908 | Lys50Thr | *phtE* | Pneumococcal histidine triad protein |
| 896300 | **A to C** | spr0908 | Glu387Asp | *phtE* | Pneumococcal histidine triad protein |
| 952093 | C to T | spr0969 | Thr319Ile | *nikS* | Nikkomycin biosynthesis protein |
| 1126380 | C to T | spr1125 |  |  | Conserved hypothetical protein |
| **Lineage 5** | | | | | |
| 152821 | **T to G** | spr0144 | Leu164* |  | Conserved hypothetical protein |
| 188183 | **T to G** | spr0180 | Phe152Val | *cls* | Cardiolipin synthase |
| 601550 | **A to C** | spr0585 | Lys115Thr | *thyA* | Thymidylate synthase |
| 1537663 | **T to G** | spr1556 | Lys199Gln | *dnaB* | Chromosome replication initiation protein/membrane attachment |
| 1642851 | **A to C** | spr1669 | Lys307Gln | *galR* | Transcriptional regulator |
| **Lineage 7** | | | | | |
| 165644 | **A to C** | spr0157 | Tyr290Asp |  | Conserved hypothetical protein |
| 437429 | C to T | spr0438 | Ser136Phe | *pyrG* | CTP synthetase |
| 788643 | **T to G** | spr0791 | Ile128Arg | *hsdS* | Type I restriction-modification enzyme, S subunit |
| 1868861 | **T to G** | spr1889 | Thr15Pro | *argR* | Arginine repressor |
| **Lineage 9** | | | | | |
| 359472 | **T to G** | spr0360 | *53Gly |  | Conserved hypothetical protein |
| 662408 | **T to G** | spr0658 |  |  | Hypothetical protein |
| 1096106 | G to A | spr1099 | Ala609Val | *gyrA* | DNA gyrase subunit A |
| 1164974 | C to T | spr1162 | Arg194Gln | *htpX* | Heat shock protein |
| 1587881 | G to T | spr1613 | Leu54Met |  | Hypothetical protein |
| **Lineage 11** | | | | | |
| 92172 | **A to C** | spr0084 |  |  | Conserved hypothetical protein |
| 92776 | G to T | spr0085 | Arg48Ser |  | Hypothetical protein |
| 461069 | **A to C** | spr0454 | Lys14Gln | *grpE* | Heat shock protein |
| 721966 | **A to C** | spr0720 | Ser77Ala |  | Hypothetical protein |
| **Lineage 13** | | | | | |
| 496834 | C to T | spr0496 |  |  | IS861-truncation |
| 975436 | **T to G** | spr0990 |  |  | Conserved hypothetical protein, similar to alanine acetyl transferase |
| 1111312 | T to C | spr1109 | Ile76Val | *fhs* | Formate-tetrahydrofolate ligase |
| 1814595 | C to A | none |  |  |  |
| 2027275 | **T to G** | spr2035 |  |  | ABC transporter, ATP-binding protein |
| **Lineage 15** | | | | | |
| 287521 | G to A | spr0286 | Ala870Thr | *hysA* | Hyaluronate lyase precursor |
| 311238 | **T to G** | spr0310 | Phe191Cys | *dexB* | α-1,6-glucosidase |
| 316136 | **A to C** | spr0316 | Glu141Ala | *cps2I* | Synthesis of type 2 capsular polysaccharide |
| 615237 | C to T | spr0602 |  |  | ABC transporter, ATP-binding protein |
| 726311 | **T to G** | none |  |  |  |
| 1161593 | **A to C** | spr1157 |  | *nth* | Endonuclease III |
| 1393161 | **T to G** | spr1409 | Glu191Asp |  | Conserved hypothetical protein |
| 1414908 | T to C | spr1431 | Asp370Gly | *lytC* | 1,4-β-N-acetylmuramidase |
| 1800278 | A to G | spr1825 |  | *gapA* | Glyceraldehyde-3-phosphate dehydrogenase (phosphorylating) |
| **Lineage 17** | | | | | |
| 95571 | **A to C** | spr0087 | Ile79Met |  | Hypothetical protein |
| 115035 | C to T | spr0106 |  |  | Transporter truncation |
| 1111312 | T to C | spr1109 | Ile76Val | *fhs* | Formate-tetrahydrofolate ligase |
| 1396511 | A to G | none |  |  |  |
| **Lineage 19** | | | | | |
| 742872 | **A to C** | spr0747 |  |  | Conserved hypothetical protein |
| 1462432 | **A to C** | none |  |  |  |
| **Lineage 21** | | | | | |
| 1003834 | C to A | spr1024 | Leu117Met | *ligA* | DNA ligase |
| **Lineage 23** | | | | | |
| 267635 | G to A | spr0265 | Gly150Glu |  | Conserved hypothetical protein |
| 465446 | **T to G** | spr0456 | Leu269Arg | *dnaJ* | Heat shock protein |
| 1873919 | G to A | spr1894 | Glu348Lys | *pnpS* | Histidine kinase HK04 |

*^a^*Changes in bold font represent AT to CG transversions, which are promoted by loss of *mutX*.
